# Supplementary material for: Impaired Plakophilin-2 in obesity breaks cell cycle dynamics to breed adipocyte senescence
Source: Nat Commun. 2023 Aug 22;14:5106. doi: 10.1038/s41467-023-40596-0 (PMC10444784; doi:10.1038/s41467-023-40596-0)
Supplement: Supplementary file 1 — Supplementary Information [file 41467_2023_40596_MOESM1_ESM.pdf]

SUPPLEMENTARY INFORMATION

Impaired Plakophilin-2 in obesity breaks cell cycle dynamics to breed adipocyte senescence

Aina Lluch[1,2], Jessica Latorre[1,2], Angela Serena-Maione[3], Isabel Espadas[4], Estefanía Caballano-Infantes[1,2], José M. Moreno-Navarrete[1,2], Núria Oliveras-Cañellas[1,2], Wifredo Ricart[1,2], María M. Malagón[2,5], Alejandro Martin-Montalvo[4], Walter Birchmeier[6], Witold Szymanski[7], Johannes Graumann[7], María Gómez-Serrano[8], Elena Sommariva[3], José M. Fernández-Real[1,2,9,10], Francisco J. Ortega[1,2,10]*

[1] Department of Diabetes, Endocrinology and Nutrition, Institut d'Investigació Biomèdica de Girona (IDIBGI), Girona (Spain).
[2] CIBER de la Fisiopatología de la Obesidad y la Nutrición (CIBEROBN), Instituto de Salud Carlos III (ISCIII), Madrid (Spain).
[3] Unit of Vascular Biology and Regenerative Medicine, Centro Cardiologico Monzino IRCCS, Milan (Italy).
[4] Centro Andaluz de Biología Molecular y Medicina Regenerativa (CABIMER), Consejo Superior de Investigaciones Científicas (CSIC), University Pablo de Olavide, Seville (Spain).
[5] Department of Cell Biology, Physiology and Immunology, Instituto Maimonides de Investigación Biomédica de Cordoba (IMIBIC), University of Cordoba, Reina Sofia University Hospital, Cordoba (Spain).
[6] Max Delbrück Center for Molecular Medicine, Berlin-Buch (Germany).
[7] Institute of Translational Proteomics, Biochemical/Pharmacological Centre, Philipps University, Marburg (Germany).
[8] Institute for Tumor Immunology, Center for Tumor Biology and Immunology, Philipps University, Marburg (Germany)
[9] Department of Medical Sciences, School of Medicine, University of Girona (Spain).
[10] These authors jointly supervised this work.

*Corresponding author:
Francisco J. Ortega. Email: fortega@idibgi.org

Short title: Relevance of Plakophilin-2 in adipocytes.

SUPPLEMENTARY TABLES

Supplementary Table S1. Anthropometric and biochemical data of 24 health-weight age-matched women selected as a reference group (controls) for cross-sectional comparisons, and 20 obese women before (baseline) and ~2 years after bariatric surgery (post-weight loss). See also in Figure 2b.

Study variables	Controls (n=24)	Obese baseline (n=20)	Obese post-weight loss	p-value[a]	p-value[b]
Age (years)	45 ± 5	49 ± 9	51 ± 9	0.105	<0.0001
BMI (kg/m^2)	24.4 ± 2.9	43.1 ± 5	29.2 ± 5.5	<0.0001	<0.0001
Fat mass (%)	38.6 ± 5	56.1 ± 7.4	40.1 ± 7.4	<0.0001	<0.0001
SBP (mmHg)	116.4 ± 13.7	128.8 ± 13.7	130.7 ± 16.9	0.003	0.215
DBP (mmHg)	70.8 ± 12.2	79 ± 9.7	74.2 ± 12.6	0.014	0.089
Glucose (mg/dl)	89.6 ± 9.5	94.5 ± 14	87.4 ± 13.5	0.162	0.018
HbA1c (%)	5.4 ± 0.3	5.5 ± 0.6	5.3 ± 0.3	0.466	0.261
Cholesterol (mg/dl)	188.1 ± 35.9	183.1 ± 33.6	184.7 ± 51.8	0.624	0.851
LDL (mg/dl)	111.4 ± 31.8	105.1 ± 28.8	100.4 ± 29.7	0.477	0.407
HDL (mg/dl)	58.8 ± 13.2	56.5 ± 13.1	73.8 ± 22.1	0.547	<0.0001
Triglycerides (mg/dl)	94.6 ± 46.3	107.9 ± 43.1	81.4 ± 28.7	0.31	0.006
PKP2 in SC	0.0197 ± 0.0213	0.0032 ± 0.0036	0.024 ± 0.0183	<0.0001	<0.0001

Values represent mean ± S.D. BMI: body mass index, S/DBP: systolic/diastolic blood pressure, HbA1c: glycated haemoglobin, L/HDL: low/high density proteins, PKP2: Plakophilin-2, SC: subcutaneous adipose tissue. [a]Results in controls (BMI<30 kg/m^2) versus severe obese (BMI≥35 kg/m^2) women (baseline) were compared by two-tailed Fisher's exact t-test. [b]Results post-weight loss versus baseline were compared by paired t-test.

Supplementary Table S2. Characteristics of study subjects, including gene expression measures taken in depths of subcutaneous and omental adipose tissue. See also in Figures 2d and 2f.

	NO with NGT	NO with IGT	Ob with NGT	Ob with IGT	ANOVA	p-value[a]	p-value[b]	p-value[c]
n (men/women)	19/77	3/8	18/53	8/34				
Age (years)	48 ± 10	55 ± 11	44 ± 11	46 ± 10	0.01	0.183	0.128	0.846
BMI (kg/m^2)	24.9 ± 3.5	27.8 ± 1.7	43.7 ± 7.5	45.1 ± 7.9	<0.0001	0.428	<0.0001	0.63
Fat mass (%)	33.4 ± 6.4	37.6 ± 6.4	54.4 ± 9.8	57.2 ± 11.7	<0.0001	0.437	<0.0001	0.382
SBP (mmHg)	123 ± 15	137 ± 19	135 ± 17	137 ± 21	<0.0001	0.047	<0.0001	0.95
DBP (mmHg)	73 ± 12	84 ± 8	80 ± 11	80 ± 11	<0.0001	0.02	0.002	1
Fasting glucose (mg/dl)	88.1 ± 8.8	137.7 ± 53.6	90.5 ± 8.8	123.9 ± 40.1	<0.0001	<0.0001	0.905	<0.0001
Hb1Ac (%)	5.3 ± 0.41	7.21 ± 2.93	5.04 ± 0.48	5.67 ± 1.36	<0.0001	<0.0001	0.475	0.027
Cholesterol (mg/dl)	193.5 ± 40.2	239.8 ± 40.3	192.1 ± 38.2	185.8 ± 33.7	0.001	0.002	0.996	0.85
LDL (mg/dl)	113 ± 32.9	159.1 ± 34.2	114.9 ± 32.9	109.5 ± 32.2	<0.0001	<0.0001	0.985	0.872
HDL (mg/dl)	63.3 ± 20.2	48.4 ± 8.4	58.6 ± 42.7	50.6 ± 11.1	0.083	0.388	0.746	0.517
Triglycerides (mg/dl)	95.3 ± 45.8	174.5 ± 74.7	113.8 ± 69	138.4 ± 68.7	<0.0001	0.001	0.226	0.183
Gene expression in SC adipose tissue								
PKP2	0.0218 ± 0.0252	0.0084 ± 0.0067	0.0066 ± 0.008	0.0062 ± 0.0076	<0.0001	0.153	<0.0001	1
LEP	0.481 ± 0.309	0.816 ± 0.179	0.842 ± 0.367	0.944 ± 0.576	<0.0001	0.083	<0.0001	0.724
HSD11B1	0.0406 ± 0.0379	0.0573 ± 0.052	0.0729 ± 0.0447	0.0711 ± 0.0441	<0.0001	0.947	<0.0001	0.997
SPP1	0.0294 ± 0.064	0.0727 ± 0.05	0.0746 ± 0.0861	0.1198 ± 0.1907	0.006	0.886	0.169	0.235
SLC27A2	0.00193 ± 0.00324	0.00031 ± 0.00055	0.00022 ± 0.00066	0.00011 ± 0.00032	<0.0001	0.387	<0.0001	0.994
FASN	0.91 ± 0.859	0.299 ± 0.434	0.383 ± 0.664	0.144 ± 0.174	<0.0001	0.056	<0.0001	0.491
TNF	0.00238 ± 0.00263	0.00247 ± 0.00083	0.00316 ± 0.00209	0.00353 ± 0.00255	0.119	1	0.347	0.905
TMEM26	0.00062 ± 0.00074	0.00046 ± 0.00007	0.00068 ± 0.00083	0.00085 ± 0.00134	0.715	0.987	0.989	0.866
NRG4	0.00017 ± 0.00015	0.00013 ± 0.00009	0.00033 ± 0.00059	0.00047 ± 0.00133	0.351	1	0.679	0.843
Gene expression in OM adipose tissue								
PKP2	0.0245 ± 0.0247	0.0132 ± 0.0065	0.0266 ± 0.023	0.028 ± 0.0219	0.418	0.196	0.969	0.995
LEP	0.189 ± 0.162	0.322 ± 0.114	0.324 ± 0.193	0.256 ± 0.099	0.008	0.246	0.008	0.742
HSD11B1	0.0399 ± 0.0327	0.0899 ± 0.0788	0.0539 ± 0.0254	0.0502 ± 0.0276	0.027	0.091	0.099	0.946
SPP1	0.0382 ± 0.0483	0.1273 ± 0.1104	0.0581 ± 0.1153	0.0615 ± 0.0513	0.376	0.3	0.872	0.999
SLC27A2	0.00376 ± 0.00511	0.00023 ± 0.00018	0.00073 ± 0.00226	0.00067 ± 0.00184	0.004	0.198	0.004	1
FASN	0.541 ± 0.58	0.157 ± 0.099	0.101 ± 0.123	0.057 ± 0.056	<0.0001	0.034	<0.0001	0.954
TNF	0.00311 ± 0.00322	0.00192 ± 0.00161	0.00633 ± 0.00943	0.00537 ± 0.00293	0.04	0.953	0.052	0.948
TMEM26	0.00185 ± 0.00146	0.00198 ± 0.00166	0.00305 ± 0.00197	0.00289 ± 0.00166	0.004	0.998	0.004	0.98
NRG4	0.00223 ± 0.00262	0.0013 ± 0.00149	0.00375 ± 0.00257	0.00475 ± 0.00267	0.001	0.961	0.034	0.367

Values represent mean ± S.D. NO/Ob: non-obese/obese participants, N/IGT: normal/ impaired glucose tolerance, BMI: body mass index, S/DBP: systolic/diastolic blood pressure, HbA1c: glycated haemoglobin, L/HDL: low/high density proteins, SC/OM: subcutaneous/omental, *PKP2*: plakophilin-2, *LEP*: leptin, *HSD11B1*: hydroxysteroid 11-beta dehydrogenase 1, *SPP1*: secreted phosphoprotein 1 (aka osteopontin), *SLC27A2*: solute carrier family 27 member 2, *FASN*: fatty acid synthase, *TNF*: tumor necrosis factor, *TMEM26*: transmembrane protein 26, *NRG4*: neuregulin-4. One-way ANOVA was followed by Tukey's honestly significant difference (HSD) post-hoc test for [a]non-obese subjects with and without IGT, [b]non-obese participants *versus* obese subjects without IGT, and [c]obese participants with and without IGT. Obesity was set at BMI≥30 kg/m^2. IGT was defined as impaired fasting glucose (≥110 mg/dl), and/or HbA1c of ≥5.7%.

Supplementary Table S3. Spearman's rho tests for correlations between study variables and measures of *PKP2* gene expression taken in bulk subcutaneous and omental adipose tissue. See also in Figures 2e and 2g, and in Supplementary Figures S2f-i.

	PKP2 in OM (n=131)		*PKP2* in SC (n=183)	
Correlations	r	p	r	p
Age (years)	-0.28	0.001	0.04	0.578
BMI (kg/m^2)	0.13	0.146	-0.49	<0.0001
Fat mass (%)	0.11	0.235	-0.48	<0.0001
SBP (mmHg)	-0.13	0.194	-0.19	0.014
DBP (mmHg)	-0.02	0.828	-0.11	0.186
Fasting glucose (mg/dl)	-0.17	0.062	-0.23	0.002
Hb1Ac (%)	-0.39	<0.0001	0.01	0.924
Cholesterol (mg/dl)	-0.26	0.005	-0.14	0.062
LDL (mg/dl)	-0.29	0.002	-0.1	0.214
HDL (mg/dl)	0.07	0.489	0.11	0.134
Triglycerides (mg/dl)	-0.15	0.106	-0.31	<0.0001
LEP in OM/SC	-0.35	0.002	-0.52	<0.0001
SPP1 in OM/SC	-0.25	0.026	-0.45	<0.0001
HSD11B1 in OM/SC	-0.32	0.001	-0.32	<0.0001
SLC27A2 in OM/SC	0.29	0.007	0.51	<0.0001
PKP2 in SC (n=90 paired samples)	0.06	0.544		

OM/SC: omental/subcutaneous, BMI: body mass index, S/DBP: systolic/diastolic blood pressure, HbA1c: glycated haemoglobin, L/HDL: low/high density proteins, *LEP*: leptin, *HSD11B1*: hydroxysteroid 11-beta dehydrogenase 1, *SPP1*: secreted phosphoprotein 1 (also known as osteopontin), *SLC27A2*: solute carrier family 27 member 2, *PKP2*: plakophilin-2.

Supplementary Table S4. Functional enrichment of proteins down (in blue; query size of 488) and upregulated (in red; 1,113) in si-PKP2 adipocytes (first) and preadipocytes (589 and 801, respectively; second table). Enrichment analyses were performed with g:Profiler (https://biit.cs.ut.ee/gprofiler/gost). Only relevant terms in the Gene Ontology (GO) database are listened below and spotlighted in Supplemental Figure S5a-d. Abundance changes affecting proteins annotated in categories of interest are represented in Figure 4h.

#	source	term_name	term_id	adjusted_p-value	term_size	intersection_size
1	GO:MF	protein binding	GO:0005515	2,11E-21	14881	447
2	GO:MF	catalytic activity	GO:0003824	5,84E-15	5758	227
3	GO:MF	enzyme regulator activity	GO:0030234	1,21E-03	1300	59
4	GO:MF	translation factor activity, RNA binding	GO:0008135	2,56E-03	99	12
5	GO:MF	single-stranded DNA helicase activity	GO:0017116	7,39E-03	23	6
6	GO:MF	linear malto-oligosaccharide phosphorylase activity	GO:0102250	7,54E-03	3	3
7	GO:MF	SHG alpha-glucan phosphorylase activity	GO:0102499	7,54E-03	3	3
8	GO:MF	glycogen phosphorylase activity	GO:0008184	7,54E-03	3	3
9	GO:MF	extracellular matrix structural constituent	GO:0005201	1,20E-02	174	15
10	GO:MF	P-type calcium transporter activity	GO:0005388	2,07E-02	9	4
11	GO:MF	structural constituent of postsynaptic actin cytoskeleton	GO:0098973	2,96E-02	4	3
12	GO:BP	organonitrogen compound metabolic process	GO:1901564	2,48E-15	6441	239
13	GO:BP	organelle organization	GO:0006996	8,19E-07	3608	137
14	GO:BP	cell cycle	GO:0007049	1,07E-06	1825	84
15	GO:BP	double-strand break repair via break-induced replication	GO:0000727	2,86E-04	12	6
16	GO:BP	regulation of DNA-templated DNA replication initiation	GO:0030174	1,46E-03	15	6
17	GO:BP	biological process involved in interspecies interaction between organisms	GO:0044419	2,00E-03	1752	72
18	GO:BP	mesenchyme migration	GO:0090131	3,32E-03	5	4
19	GO:BP	response to virus	GO:0009615	5,86E-03	403	26
20	GO:BP	actin filament-based process	GO:0030029	5,90E-03	817	41
21	GO:BP	cytoplasmic translational initiation	GO:0002183	7,16E-03	40	8
22	GO:BP	collagen metabolic process	GO:0032963	1,04E-02	103	12
23	GO:BP	DNA unwinding involved in DNA replication	GO:0006268	1,90E-02	22	6
24	GO:BP	locomotion	GO:0040011	2,01E-02	1365	57
25	GO:BP	cell motility	GO:0048870	2,28E-02	1705	67
26	GO:BP	non-membrane-bounded organelle assembly	GO:0140694	3,02E-02	389	24
27	GO:BP	growth	GO:0040007	3,12E-02	937	43
28	GO:CC	cytoplasm	GO:0005737	1,07E-34	12365	404
29	GO:CC	focal adhesion	GO:0005925	9,33E-11	425	38
30	GO:CC	MCM complex	GO:0042555	1,53E-05	11	6
31	GO:CC	collagen-containing extracellular matrix	GO:0062023	3,76E-05	430	29
32	GO:CC	supramolecular complex	GO:0099080	1,21E-04	1411	61
33	GO:CC	chromosomal region	GO:0098687	2,84E-04	396	26
34	GO:CC	midbody	GO:0030496	1,22E-03	207	17
35	GO:CC	MHC class I protein complex	GO:0042612	4,96E-03	8	4
36	GO:CC	cell division site	GO:0032153	1,04E-02	73	9
1	GO:MF	protein binding	GO:0005515	5,10E-40	14881	999
2	GO:MF	structural constituent of chromatin	GO:0030527	1,06E-14	101	33
3	GO:MF	catalytic activity	GO:0003824	2,26E-13	5758	439
4	GO:MF	structural constituent of cytoskeleton	GO:0005200	5,30E-11	109	30
5	GO:MF	P-type ion transporter activity	GO:0015662	4,39E-03	21	8
6	GO:MF	structural constituent of muscle	GO:0008307	7,56E-03	43	11
7	GO:MF	magnesium ion binding	GO:0000287	1,08E-02	227	29
8	GO:BP	organelle organization	GO:0006996	1,81E-27	3608	336
9	GO:BP	translation	GO:0006412	2,03E-17	745	104
10	GO:BP	ribosome biogenesis	GO:0042254	5,00E-10	312	51
11	GO:BP	organic acid metabolic process	GO:0006082	7,13E-09	979	103
12	GO:BP	nucleosome organization	GO:0034728	8,88E-08	143	30
13	GO:BP	cellular response to stress	GO:0033554	1,61E-07	1949	165
14	GO:BP	regulation of mRNA metabolic process	GO:1903311	5,01E-05	306	41
15	GO:BP	cellular respiration	GO:0045333	9,74E-05	244	35
16	GO:BP	regulation of cellular component biogenesis	GO:0044087	3,87E-04	971	88
17	GO:BP	cell morphogenesis	GO:0000902	3,60E-03	978	85
18	GO:BP	viral genome replication	GO:0019079	4,42E-03	126	21
19	GO:BP	viral release from host cell	GO:0019076	5,13E-03	31	10
20	GO:BP	regulation of binding	GO:0051098	8,33E-03	370	41
21	GO:BP	protein polymerization	GO:0051258	1,34E-02	287	34
22	GO:BP	response to hormone	GO:0009725	1,35E-02	875	76
23	GO:BP	axo-dendritic transport	GO:0008088	2,20E-02	78	15
24	GO:BP	intracellular signal transduction	GO:0035556	2,61E-02	2669	185
25	GO:BP	cell migration	GO:0016477	2,77E-02	1505	115
26	GO:BP	macromolecule modification	GO:0043412	4,00E-02	3840	251
27	GO:BP	system development	GO:0048731	4,39E-02	3987	259
28	GO:BP	cellular response to external stimulus	GO:0071496	4,57E-02	330	36
29	GO:CC	cytoplasm	GO:0005737	1,02E-83	12365	924

30	GO:CC	nucleosome	GO:0000786	2,60E-14	135	36
31	GO:CC	cytoplasmic stress granule	GO:0010494	1,75E-03	85	16
32	GO:CC	filopodium	GO:0030175	2,41E-03	107	18
33	GO:CC	preribosome	GO:0030684	8,86E-03	76	14
34	GO:CC	cytoplasmic side of membrane	GO:0098562	1,81E-02	207	25
35	GO:CC	polysome	GO:0005844	2,15E-02	72	13
36	GO:CC	myosin II filament	GO:0097513	4,96E-02	3	3

#	source	term_name	term_id	adjusted_p_value	term_size	intersection_size
1	GO:MF	catalytic activity	GO:0003824	6,13E-21	5758	281
2	GO:MF	protein binding	GO:0005515	7,04E-21	14881	530
3	GO:MF	P-type calcium transporter activity	GO:0005388	3,09E-07	9	7
4	GO:MF	structural constituent of cytoskeleton	GO:0005200	8,97E-06	109	17
5	GO:MF	double-stranded RNA binding	GO:0003725	1,43E-04	76	13
6	GO:MF	poly-purine tract binding	GO:0070717	1,02E-02	30	7
7	GO:MF	phenanthrene 9,10-monooxygenase activity	GO:0018636	1,29E-02	3	3
8	GO:MF	peptide antigen binding	GO:0042605	2,45E-02	34	7
9	GO:MF	alcohol dehydrogenase (NADP+) activity	GO:0008106	2,69E-02	24	6
10	GO:MF	poly(U) RNA binding	GO:0008266	3,45E-02	25	6
11	GO:BP	response to stress	GO:0006950	8,95E-25	4017	222
12	GO:BP	small molecule metabolic process	GO:0044281	2,19E-08	1865	102
13	GO:BP	phosphorus metabolic process	GO:0006793	4,07E-06	2788	128
14	GO:BP	organelle organization	GO:0006996	4,94E-04	3608	147
15	GO:BP	antigen proc. and pres. of exogenous peptide antigen via MHC class Ib	GO:0002477	1,33E-03	4	4
16	GO:BP	cell cycle	GO:0007049	2,14E-03	1825	85
17	GO:BP	cell development	GO:0048468	5,77E-03	2763	115
18	GO:BP	regulation of cardiac conduction	GO:1903779	8,39E-03	25	7
19	GO:BP	generation of precursor metabolites and energy	GO:0006091	1,81E-02	449	30
20	GO:BP	protection from natural killer cell mediated cytotoxicity	GO:0042270	1,91E-02	6	4
21	GO:BP	T cell mediated immunity	GO:0002456	1,97E-02	108	13
22	GO:BP	homeostatic process	GO:0042592	2,14E-02	1673	76
23	GO:BP	neurogenesis	GO:0022008	4,14E-02	1705	76
24	GO:CC	cytoplasm	GO:0005737	7,09E-58	12365	507
25	GO:CC	catalytic complex	GO:1902494	2,92E-07	1792	91
26	GO:CC	MHC class I protein complex	GO:0042612	2,80E-06	8	6
27	GO:CC	cytoskeleton	GO:0005856	9,07E-04	2412	100
28	GO:CC	NF-kappaB complex	GO:0071159	2,20E-03	6	4
29	GO:CC	I-kappaB/NF-kappaB complex	GO:0033256	2,29E-02	4	3
30	GO:CC	semaphorin receptor complex	GO:0002116	4,36E-02	11	4
31	GO:CC	membrane raft	GO:0045121	4,93E-02	324	21
1	GO:MF	protein binding	GO:0005515	1,31E-30	14881	724
2	GO:MF	extracellular matrix structural constituent	GO:0005201	2,46E-10	174	32
3	GO:MF	catalytic activity	GO:0003824	1,09E-09	5758	319
4	GO:MF	molecular adaptor activity	GO:0060090	1,65E-04	545	48
5	GO:MF	enzyme regulator activity	GO:0030234	7,84E-04	1300	87
6	GO:MF	peptidyl-lysine 5-dioxygenase activity	GO:0070815	1,83E-03	4	4
7	GO:MF	translation regulator activity	GO:0045182	1,14E-02	157	19
8	GO:MF	RNA binding	GO:0003723	1,31E-02	3113	167
9	GO:MF	heparin binding	GO:0008201	1,36E-02	173	20
10	GO:MF	phosphotransferase activity, carboxyl group as acceptor	GO:0016774	4,64E-02	3	3
11	GO:MF	protein kinase binding	GO:0019901	4,67E-02	709	50
12	GO:BP	organonitrogen compound metabolic process	GO:1901564	9,07E-23	6441	378
13	GO:BP	supramolecular fiber organization	GO:0097435	2,96E-18	819	92
14	GO:BP	ribosome biogenesis	GO:0042254	5,64E-13	312	47
15	GO:BP	basement membrane organization	GO:0071711	1,83E-04	38	11
16	GO:BP	Golgi vesicle transport	GO:0048193	1,28E-03	309	31
17	GO:BP	small molecule metabolic process	GO:0044281	2,84E-03	1865	109
18	GO:BP	Rap protein signal transduction	GO:0032486	7,74E-03	13	6
19	GO:BP	reg. of cysteine-type endopeptidase activity involved in apoptotic process	GO:0043281	1,60E-02	203	22
20	GO:BP	epithelial to mesenchymal transition	GO:0001837	1,96E-02	161	19
21	GO:BP	carbohydrate derivative metabolic process	GO:1901135	3,33E-02	1059	67
22	GO:CC	cytoplasm	GO:0005737	6,48E-63	12365	673
23	GO:CC	preribosome	GO:0030684	4,02E-06	76	16
24	GO:CC	myosin II filament	GO:0097513	1,71E-02	3	3

Supplementary Table S5. Characteristics of unrelated patients from which a subcutaneous fat biopsy was taken, during implantable cardioverter defibrillator implant, for the extraction of subcutaneous fat-derived stromal cells.

#	Patients	Age	Sex	Clinical diagnosis	Genetics
1	*PKP2*-1	27 years	W	ACM	*PKP2* c.2013delC p.Lys672ArgfsX12
2	*PKP2*-2	39 years	W	ACM	*PKP2* c.2013delC p.Lys672ArgfsX12
3	*PKP2*-3	24 years	M	ACM	*PKP2* c.121delG p.V406FfsX13
4	*PKP2*-4	42 years	M	ACM	*PKP2* c.1643delG p.Gly548ValfsX15
5	*PKP2*-5	36 years	M	ACM	*PKP2* c.2013delC p.Lys672ArgfsX12
6	Non-*PKP2*-1	29 years	W	ACM	No mutations in ACM-associated genes
7	Non-*PKP2*-2	70 years	M	ACM	No mutations in ACM-associated genes
8	Non-*PKP2*-3	52 years	M	ACM	No mutations in ACM-associated genes
9	Non-*PKP2*-4	46 years	M	MYO	-
10	Non-*PKP2*-5	69 years	M	Ischemia	-

ACM: arrhythmogenic cardiomyopathy; M: man, W: woman; MYO: myocarditis.

Supplementary Table S6. Characteristics of mice from which subcutaneous fat was taken, processed and analysed.

Genetics	Age	Sex
Pkp2$^{+/-}$ 1	5 months	F
Pkp2$^{+/-}$ 2	5 months	F
Pkp2$^{+/-}$ 3	3 months	M
Pkp2$^{+/-}$ 4	3 months	M
Pkp2$^{+/-}$ *5*	3 months	M
Wt 1	5 months	F
Wt 2	5 months	F
Wt 3	5 months	F
Wt 4	3 months	M

M: male; F: female mice.

Supplementary Table S7. Paired SYBR Green primers and TaqMan assays used during this research.

Human genes	Forward SYBR Green primers	Reverse SYBR Green primers
ACACB	CTGAGTCACGTGCATATC	ACAAGTAGGCCTTGACAG
AURKA	CCTACAAAAGAATATCACGGG	CAAGTACTTCTCTGAGCATTG
BAX	AACTGGACAGTAACATGGAG	TTGCTGGCAAAGTAGAAAAG
CCNB2	ATTTTTACAGGTTCAGCCAG	ATCTCCTCATACTTGGAAGC
CCNE1	AGACATACTTAAGGGATCAGC	CACACCTCCATTAACCAATC
CDK1	ACCTATGGAGTTGTGTATAAGG	GACTGACTATATTTGGATGACG
CDK4	AGAATCTACAGCTACCAGATG	AGAGTTTCCACAGAAGAGAG
CDKN3	GAAGAACTAAAGAGCTGTGG	TTCCATTATTTCACAGCAGC
CEBPA	AGCCTTGTTTGTACTGTATG	AAAATGGTGGTTTAGCAGAG
CREG1	AGGAGAATCCATATGCTACAC	ATTATGTGAACACAAAGGGG
DDIT4	AATGTAAGAGTAGGAAGGGG	ACAGTTCTAGATGGAAGACC
DEPDC1	GAGGTCACTGATGATACATAC	TGCAGTCTGTAAGTAAGAGG
DLGAP5	CTCTAACTGTTTGGCATGAAG	TTTCTTTCAAGTGATGGGAC
ERF	CAATTTCAACAAACTGGTGC	AGGGAAGATGAAGATGAAGAG
G0S2	TGCCACTAAGGTCATTCC	TTCACCATCTTCCCCTTG
HMGB1	TACGAAAAGGATATTGCTGC	CTCCTCTTCCTTCTTTTTCTTG
HMGB2	GAAAGCAGCTAAGCTAAAG	TTCATCTTCATCCTCTTCCTC
IMPA2	GATCATCAGAAAAGCCCTTAC	GTGATCTGTTTCTGTCACAAG
LMNB1	AAAATTCTCAGGGAGAGGAG	TGGAAAAGTTCTTCCTCAAC
MKI67	GACAGAGGTTCCTAAGAGAG	AACAATCAGATTTGCTTCCG
MT1A	AAATGCAAAGAGTGCAAATG	TTCTATATCTTCGAGCAGGG
MT1F	TCCCAGATGTAAACAGAGAG	AAAGGAATGTAGCAAATGGG
PAPPA	CATGGATCTAAATCTTGGCAG	TTTTGTATAATGCCATCGCC
PDE3B	AAATTCTGGAGGTGGAAATG	ATACTCCGTAGAGAGGAAATG
PLK4	GTGGTGAGCATACTTGATTC	GTCTATCAGCAAGAGGAAAAC
PPIA	ATGGTTCCCAGTTTTTCATC	CTCCACAATATTCATGCCTTC
SCD	GCCCCTCTACTTGGAAGACGA	AAGTGATCCCATACAGGGCTC
SERPINE	ATCCACAGCTGTCATAGTC	CACTTGGCCCATGAAAAG
SLC39A3	AGGGAAAAGCTCCAGAAG	GAAGACGGTCATGAAGAAG
SPHK1	TTCCTTGAACCATTATGCTG	GATACTTCTCACTCTCTAGGTC
SQSTM1	TGTGAATTTCCTGAAGAACG	TCGATATCAACTTCAATGCC
TOP2A	TCACAAGCAAGAAATCCAAG	ATCTTCATCTGACTCTTCCAG
Mouse genes	Forward SYBR Green primers	Reverse SYBR Green primers
Acacb	GCATGAAGGACATGTATGAG	AGGGATGTAGATGAGAATGG
G0s2	AGCTGAGGGAAGAAGAAC	TATAGCTTCACTAGCTTCCC
Pde3b	AGGATTCTCAGTCAGGTTATG	GTTGTCAAATACCAAACAGC
Ppia	ACCAAACACAAACGGTTCCC	TCCCAAAGACCACATGCTTG

Human genes	Ref. # TaqMan assays	Mouse genes	Ref. # TaqMan assays
ACLY	Hs00982738_m1	*Adipoq*	Mm00456425_m1
ADIPOQ	Hs00605917_m1	*Fasn*	Mm00772290_m1
CFB	Hs00156060_m1	*Glut4*	Mm00436615_m1
FASN	Hs01005622_m1	*Il6*	Mm00446190_m1
GLUT4	Hs00168966_m1	*Irs1*	Mm01278327_m1
HSD11B1	Hs01547870_m1	*Pkp2*	Mm00503159_m1
IL6	Hs00985639_m1	*Plin1*	Mm00558672_m1
IL8	Hs00174103_m1	*Ppia*	Mm02342430_g1
IRS1	Hs00178563_m1		
LEP	Hs00174877_m1		
NRG4	Hs00945534_m1		
PKP2	Hs00428040_m1		
PLIN1	Hs00160173_m1		
PPARG	Hs01115513_m1		
PPIA	Hs99999904_m1		
SLC27A2	Hs00186324_m1		
SPP1	Hs00959010_m1		
TMEM26	Hs00415619_m1		
TNF	Hs00174128_m1		

Figure S1 (LLuch *et al.*)

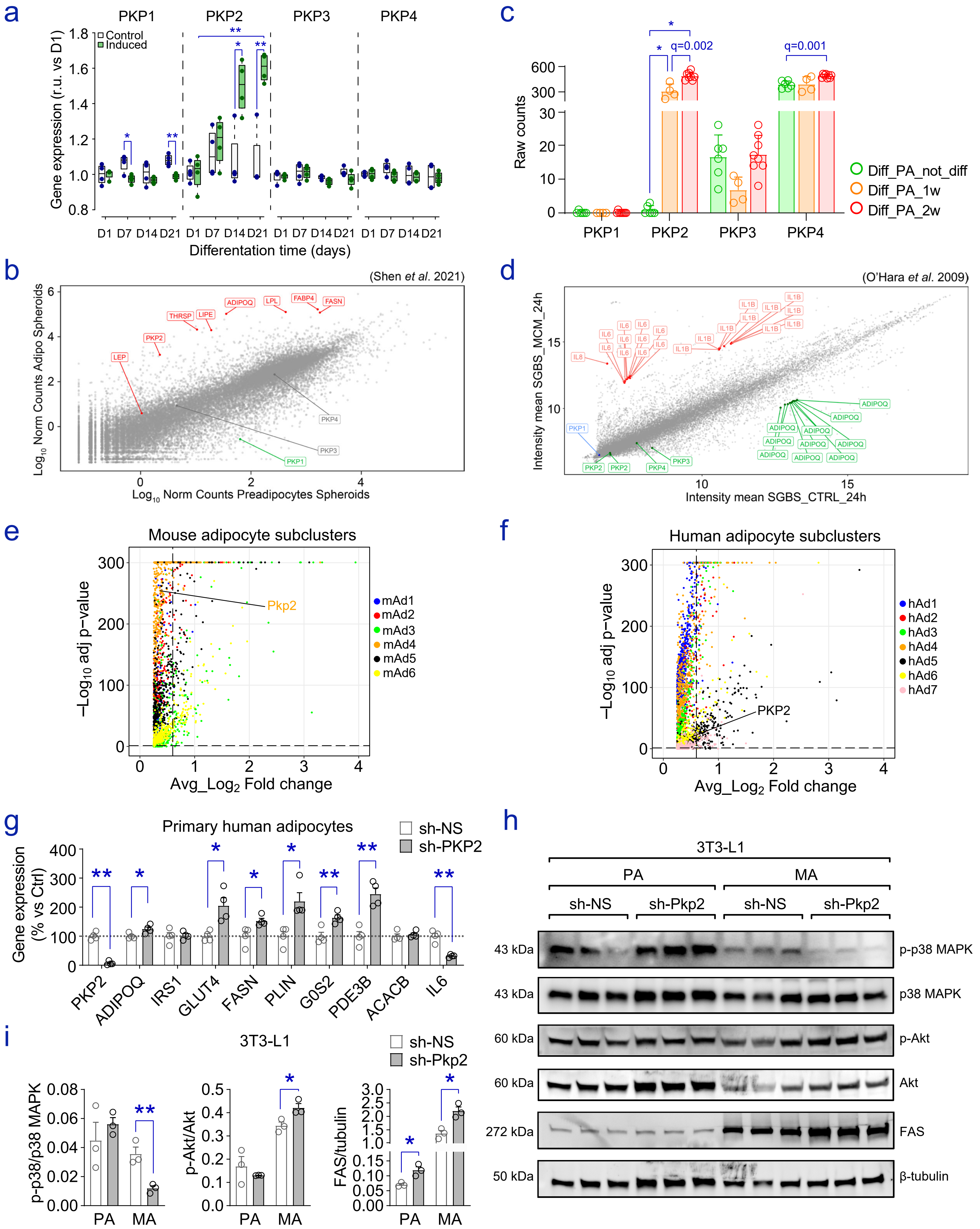

Supplementary Figure S1. (a) Plakophilin 1 to 4 (*PKP1-4*) in human fat precursor cells when induced to differentiate into adipocytes (n=4 biological replicates/group/timepoint) [1]. The black line inside the box shows the median, and the size of the box is determined by the 25th and 75th percentiles, while whiskers indicate the maximum and minimum values. (b) The scatter plot uses dots to compare gene expression values (Log_{10} normalized counts) in human preadipocytes and differentiated adipocyte spheroids [2]. Adipocyte biomarkers and *PKP1-4* are labelled in red (increased), green (decreased), or grey (no significant variations). (c) Raw counts (RNA-seq) for each plakophilin in 2D cultures of human preadipocytes (PA) under the influence of adipogenic conditions for 1 and 2 weeks (w) [2]. This plot shows the mean + S.E.M of n=6, 4, and 8 biological replicates for days 0, 7, and 14 of hormonal stimuli, respectively. *q<0.001 when comparisons were analyzed individually using the two-stage linear step-up procedure of Benjamini, Krieger and Yekutieli, with Q=1%, and without assuming a consistent S.D. (d) Expression of transcripts coding for *PKP1-4*, adiponectin (*ADIPOQ*), and pro-inflammatory adipose-related interleukins (*IL6*, *8* and *1β*) in SGBS-derived adipocytes challenged with 14% macrophage lipopolysaccharide (LPS)-conditioned media (MCM) for 24 h [3]. Red ink in labels depicts increased levels, and green labels show transcripts significantly decreased in inflamed adipocytes. Expression of *PKP2* (but none of the others plakophilins) was confirmed in (e) mouse (mAd4) and (f) human (hAd5) adipocyte subpopulations in the single-cell atlas of Emont *et al.* [4]. There, they compared the percentage of cells in the cluster where the gene is detected versus the percentage of cells on average in all the other clusters (Avg_Log_2 Fold-change). (g) Adipocyte biomarkers in differentiated human adipocytes treated with lentivirals (sh)RNAs against *PKP2* (mean + S.E.M; n=4/group). (h-i) Western plots show decreased p-p38/p38 MAPK (inflammation), and increased metabolic performance (p-Akt/Akt and FAS) in retrovirally transfected 3T3-L1 preadipocytes (PA) and mature adipocytes (MA) with diminished PKP2 (sh-Pkp2), as compared to NS controls (mean + S.E.M; n=3/group). The statistical significance for each comparison was determined by two-tailed Fisher's exact t-test. *p<0.05; **p<0.01.

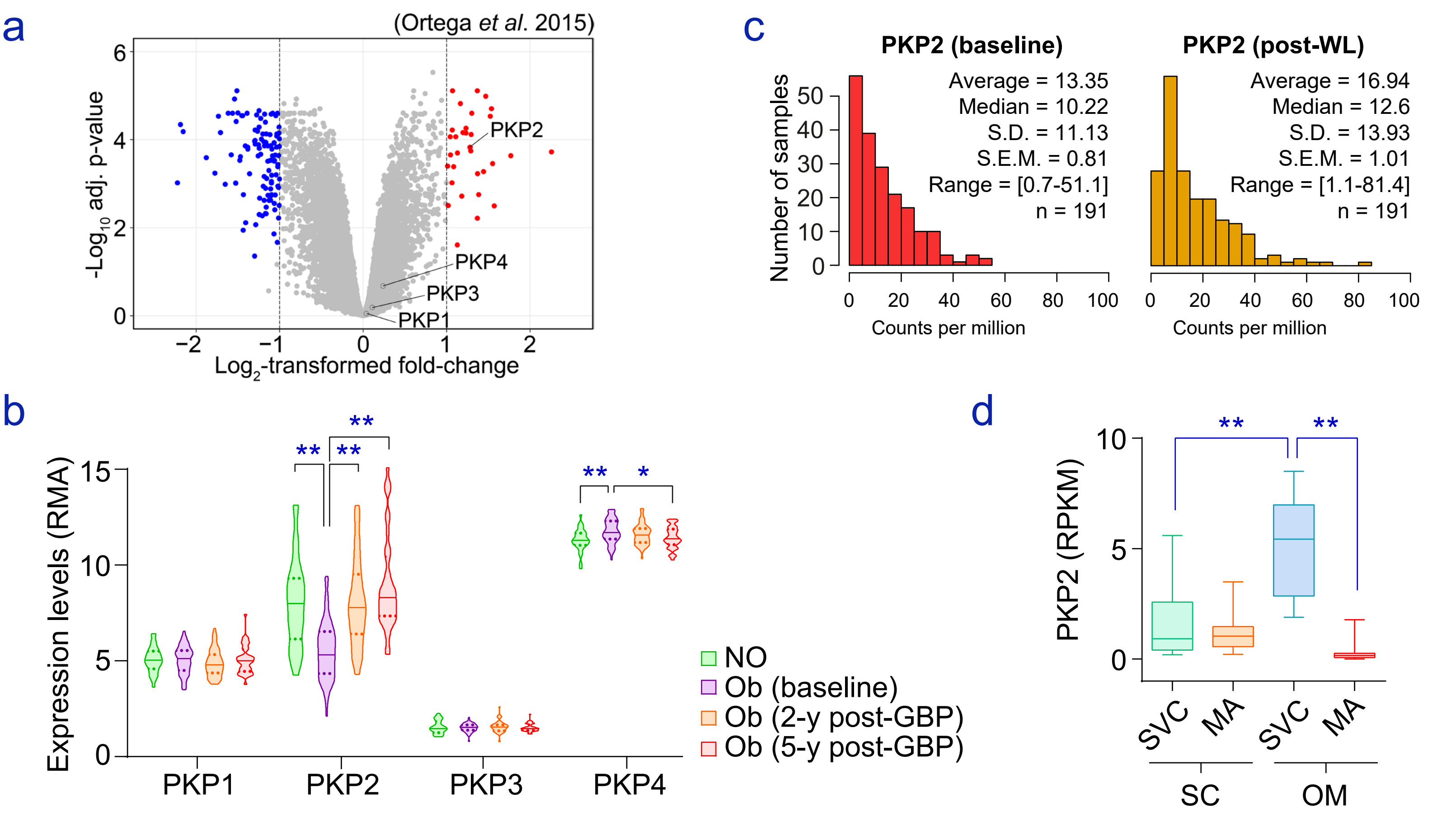

e

**PKP2** (ENSG00000057294)	Mean TPM	TPM<0.1 (%)	AC	AP	MesoC	EC	SMC	MC	NP	MastC	TC	PlasC	Cell enriched
Subcutaneous (SC) adipose tissue	7.74	0.00	0.72	-0.50	---	-0.36	-0.36	0.05	---	-0.16	-0.34	-0.23	AC
SC women	7.60	0.00	0.69	-0.59	---	-0.35	-0.46	-0.14	---	-0.29	-0.44	-0.28	AC
SC men	7.81	0.00	0.75	-0.47	---	-0.37	-0.33	0.15	---	-0.11	-0.29	-0.22	AC
Omental (OM) adipose tissue	4.25	0.00	-0.05	0.33	0.67	-0.23	-0.09	0.16	-0.08	0.33	0.19	0.16	No
OM women	3.96	0.00	-0.18	0.50	0.70	-0.09	-0.06	0.14	-0.06	0.37	0.38	0.31	MesoC
OM men	4.39	0.00	0.03	0.24	0.64	-0.32	-0.10	0.16	-0.08	0.29	0.08	0.11	---

Mean TPM	Mean expression across all samples
TPM<0.1 (%)	Percentage of samples expressing with less than 0.1 TPM expression
AC	Mean correlation values with **adipocyte** reference transcripts [ADIPOQ, LIPE, PLIN1]
AP	Mean correlation values with **adipose progenitor** reference transcripts [FKBP10, COL6A1, COL6A2]
MesoC	Mean correlation values with **mesothelial cell** reference transcripts [UPK3B, MSLN, KRT19]
EC	Mean correlation values with **endothelial cell** reference transcripts [MMRN2, ESAM, CDH5]
SMC	Mean correlation values with **smooth muscle cell** reference transcripts [KCNMB1, CNN1, MYH11]
MC	Mean correlation values with **macrophage** reference transcripts [CD68, C1QC, FCGR1G]

NP	Mean correlation values with **neutrophil** reference transcripts [CSF3R, FCGR3B, CXCR2]
MastC	Mean correlation values with **mast cell** reference transcripts [CPA3, TPSB2, TPSAB1]
TC	Mean correlation values with **T-cell** reference transcripts [TRBC2, CD6, CD3E]
PlasC	Mean correlation values with **plasma cell** reference transcripts [IGKC, JCHAIN, MZB1]
Cell enriched	Classification as cell-type enriched

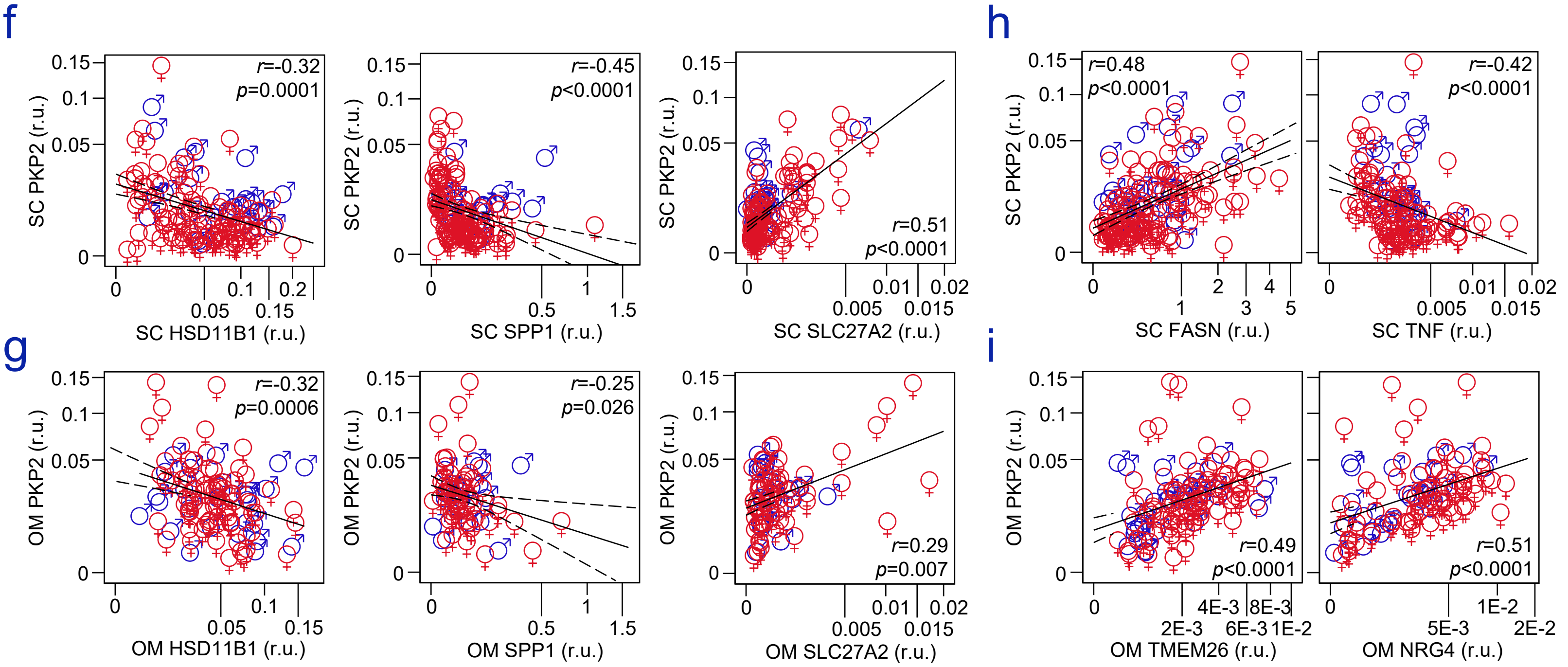

Supplementary Figure S2. (a) The Vulcano shows expression dynamics for plakophilins 1 to 4 (*PKP1-4*) in subcutaneous (SC) adipose tissue after significant weight loss [5]. (b) Also in [6], when we checked expression of SC *PKP1-4* in participants with obesity (Ob) and sex and age-matched non-obese (NO) subjects (n=28), or in the former when compared to the expression levels post-2 (n=49) and 5 (n=38) years after gastric bypass (GBP), only *PKP2* displayed a significant increase. (c) *PKP2* counts per million in samples of SC human adipose tissue before (baseline) and after diet-induced weight loss (post-WL) [7]. (d) *PKP2* gene expression levels (RPKM) in the single-cell RNA-seq of mature adipocytes (MA) and the stromal vascular cell (SVC) fraction of SC and omental (OM) adipose tissue samples [8] (see also our own results in Figure 2i). The coloured line inside the boxplot represent the median value, and the size of the box is determined by the 25th and 75th percentile, while the whiskers represents the maximum and minimum values. Statistics were performed using two-sided Student t-tests, and *$p<0.05$ and **$p<0.001$ in each dataset. (e) Mean TPM (transcript per million), detectability (TPM>0.1%), and heat map of pairwise Spearman correlation coefficients for *PKP2* (ENSG00000057294) and reference genes for different populations of adipose tissue resident cells. The lack of values for mesothelial cells (MesoC) and neutrophils (NP) in SC adipose tissue is due to the low number/absence of these cell types in this fat depot, as explained in [9]. Scatter plots show the association of 11β-hydroxysteroid dehydrogenase type 1 (*HSD11B1*), osteopontin (*SPP1*) and solute carrier family 27 (fatty acid transporter), member 2 (*SLC27A2*) with *PKP2* in (f) SC and (g) OM depots of fat (RT-PCR), and also between (h) SC *PKP2* and *FASN* (adipogenesis) and *TNF* (inflammation), and between (i) OM *PKP2* and the expression of *NRG4* and *TMEM26*. Two-sided Spearman's rank correlation coefficients (*r*) and p-values are provided for each association (see in the supplementary Table S3 and in the Source Data file provided with this article). Men (blue) and women (red) are depicted in gender glyphs.

Figure S3 (LLuch *et al.*)

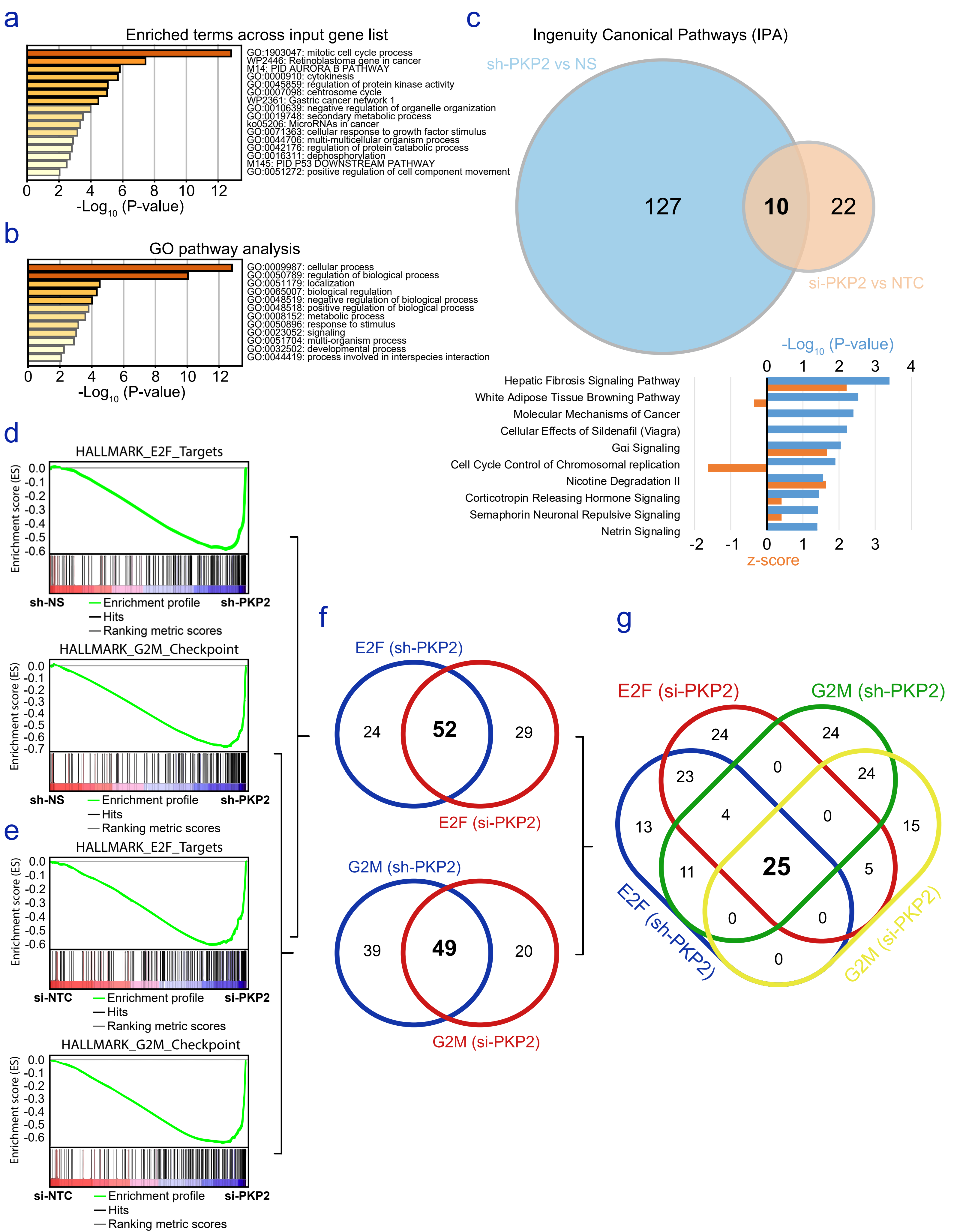

Supplementary Figure S3. (a) Enriched terms, (b) Gene Ontology (GO) pathways, and (c) Ingenuity Pathway Analysis (IPA) of changes observed in human adipocytes with diminished *PKP2* levels lasting 3 (si-PKP2) or 6 (sh-PKP2) days. The interpretation of lists of genes showing significant variations (FDR<0.05) was also performed by means of the Gene Set Enrichment Analysis (GSEA) software, based on the Hallmark gene set to depict the weight, enrichment score (ES) and profile of genes related to the biological pathways most significantly downregulated during assays lasting either (d) 6 or (e) 3 days. Venn diagrams summarize the overlap between treatments for (f) each hallmark and (g) merged E2F targets and genes involved in the G2/M checkpoint with significant variation in both experimental approaches (see also the dots inked in colours in Figure 3c).

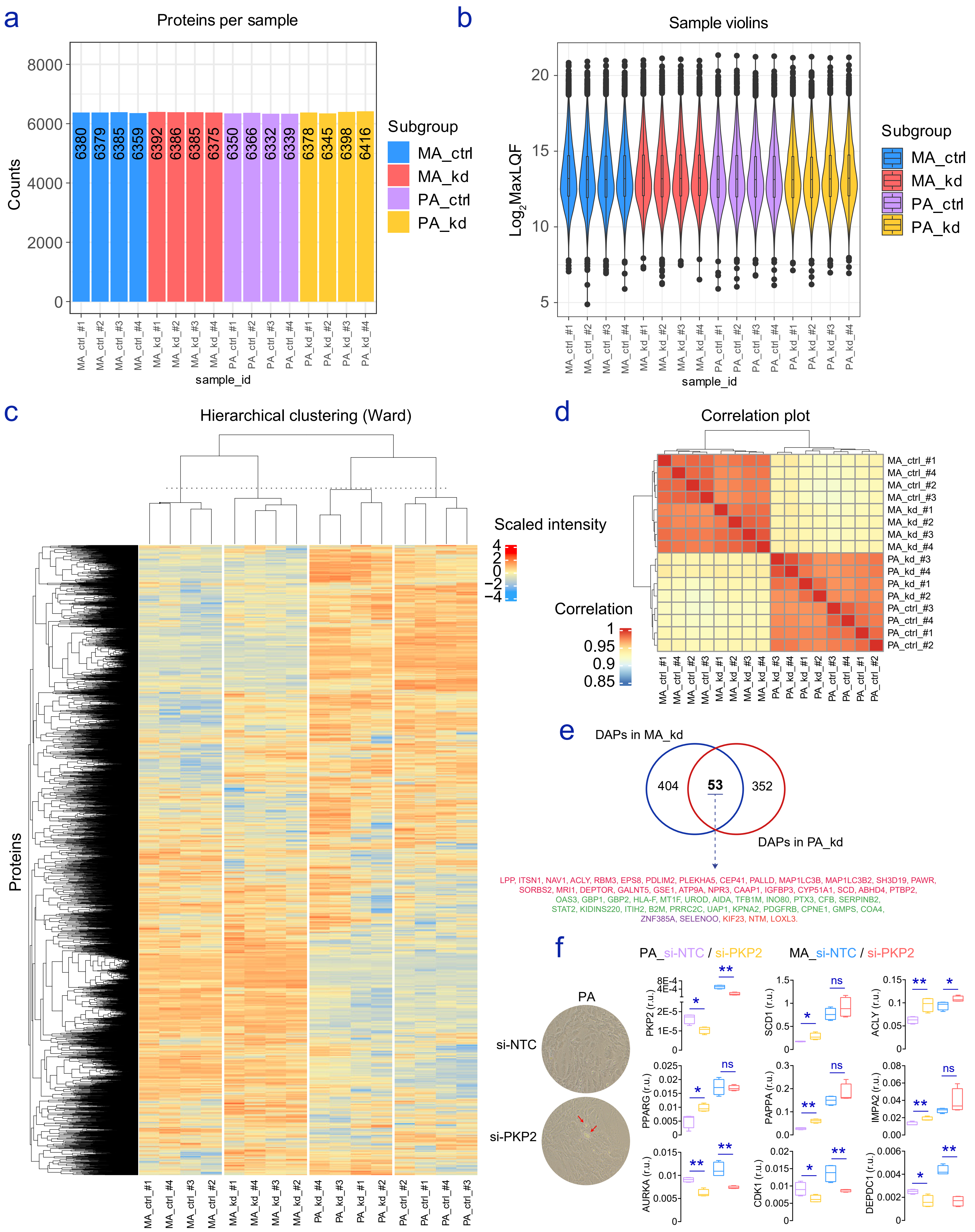
a
Proteins per sample
Counts
sample_id
Subgroup
MA_ctrl
MA_kd
PA_ctrl
PA_kd
6380 6379 6385 6359 6392 6386 6385 6375 6350 6366 6332 6339 6378 6345 6398 6416
b
Sample violins
Log2MaxLQF
c
Hierarchical clustering (Ward)
Proteins
Scaled intensity
d
Correlation plot
Correlation
e
DAPs in MA_kd
DAPs in PA_kd
404
53
352
LPP, ITSN1, NAV1, ACLY, RBM3, EPS8, PDLIM2, PLEKHA5, CEP41, PALLD, MAP1LC3B, MAP1LC3B2, SH3D19, PAWR,
SORBS2, MRI1, DEPTOR, GALNT5, GSE1, ATP9A, NPR3, CAAP1, IGFBP3, CYP51A1, SCD, ABHD4, PTBP2,
OAS3, GBP1, GBP2, HLA-F, MT1F, UROD, AIDA, TFB1M, INO80, PTX3, CFB, SERPINB2,
STAT2, KIDINS220, ITIH2, B2M, PRRC2C, UAP1, KPNA2, PDGFRB, CPNE1, GMPS, COA4,
ZNF385A, SELENOO, KIF23, NTM, LOXL3.
f
PA_si-NTC / si-PKP2
MA_si-NTC / si-PKP2
PA
si-NTC
si-PKP2
PKP2 (r.u.)
SCD1 (r.u.)
ACLY (r.u.)
PPARG (r.u.)
PAPPA (r.u.)
IMPA2 (r.u.)
AURKA (r.u.)
CDK1 (r.u.)
DEPDC1 (r.u.)

Supplementary Figure S4. (a) Raw numbers of identified proteins in each cell sample studied by quantitative proteomics and in-depth proteome-wide analysis, after filtering out peptides without replication within subgroups and proteins with less than 1 peptide. Intensities of proteins in a log_2 scale are plotted on (b) for each biological replicate and group. (c) Hierarchical clustering of proteins abundance (in rows) for each sample (in columns). (d) The cluster heatmap shows Pearson correlation coefficients between assessed protein samples. (e) The Venn diagram indicates the number of DAPs with FDR<0.05 in common for MA_kd and PA_kd (6.6% of total), which are listed below in red (increased in both), green (decreased in both), purple (increased in MA, decreased in PA), and orange (decreased in MA, increased in PA). (f) A few lipid vacuoles were apparent in the cytoplasm of si-PKP2-targeted undifferentiated PA, together with gene expression patterns reminiscent of an adipocyte-like phenotype. Box plots show the median and 25th and 75th percentiles in boxes, and whiskers that indicate the maximum and minimum values for each group (n=4/group). Statistical significance was assessed by two-tailed Fisher's exact t-test, and no adjustments were made for multiple comparisons. r.u., relative units; ns, not significant; *p<0.05; **p<0.01.

Figure S5 (LLuch *et al.*)

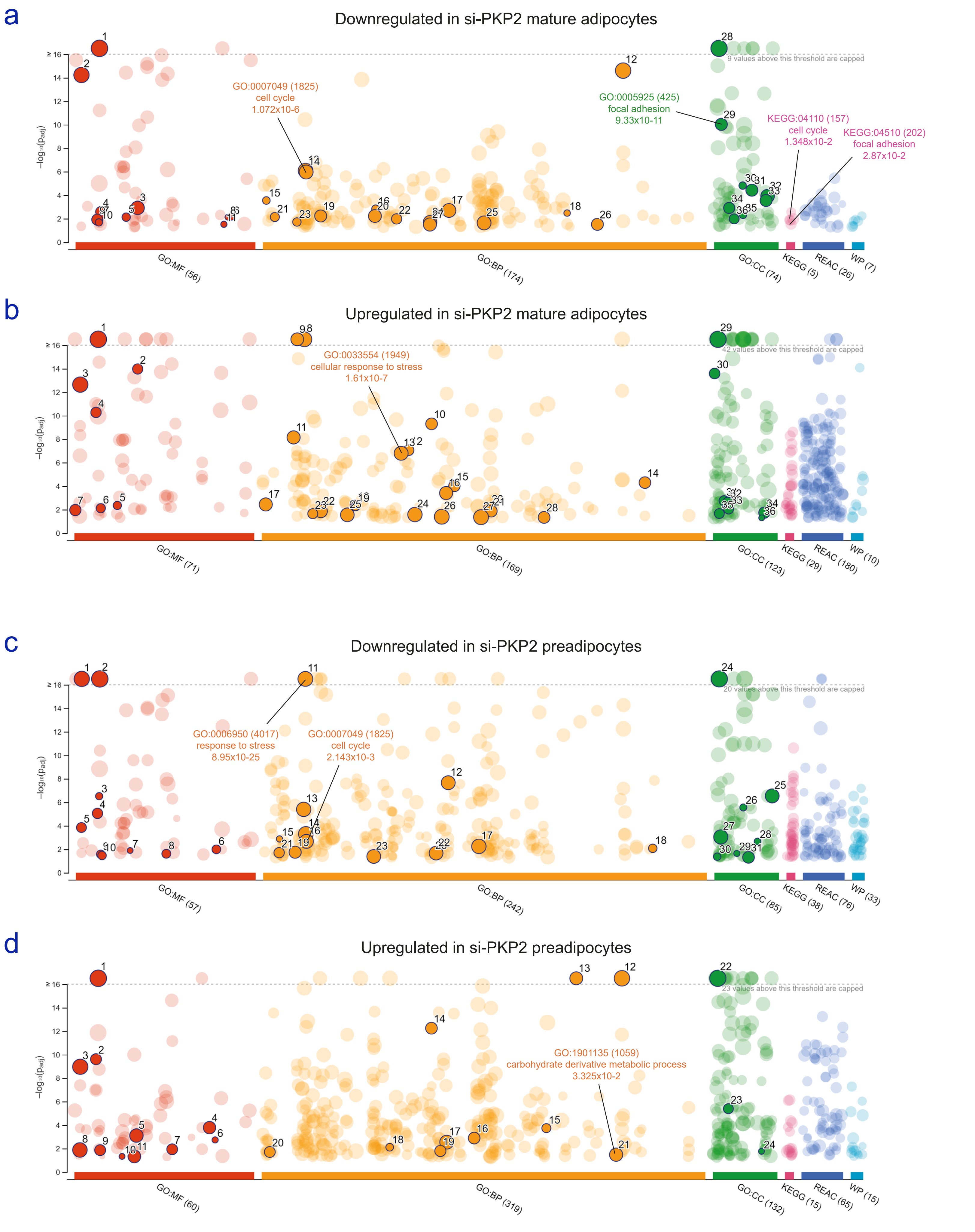

Supplementary Figure S5. Manhattan plot of significant (adjusted p-value<0.05) gene set enrichment results mapped by means of the g:Profiler tool g:GOSt (https://biit.cs.ut.ee/gprofiler/gost) on our lists of proteins (a) down and (b) upregulated in si-PKP2 adipocytes, and (c) down and (d) upregulated in si-PKP2 preadipocytes, as compared to control. Some of the most relevant terms in Gene Ontology (GO) enrichment database are labelled with reference numbers further depicted in the supplementary Table S4.

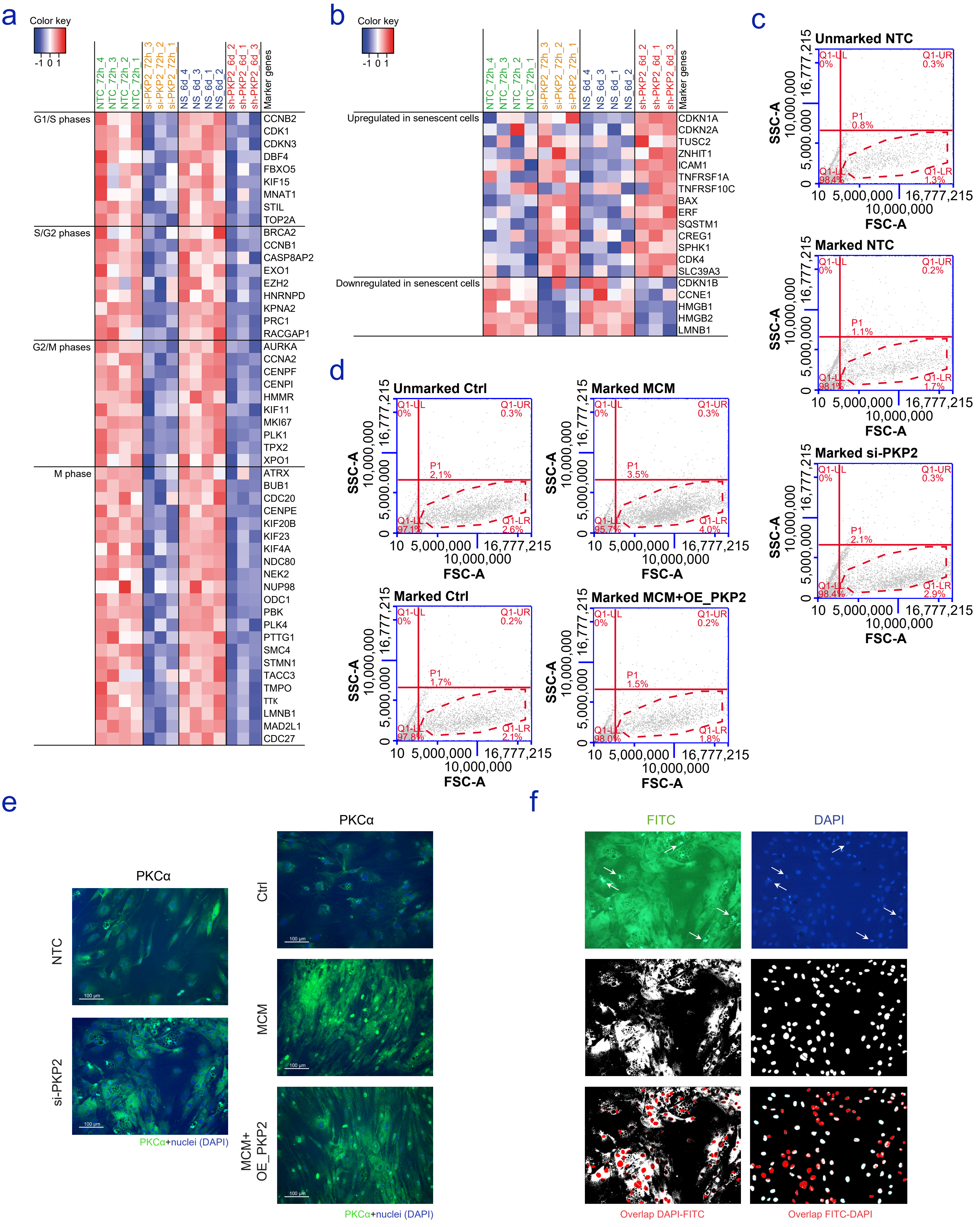
a
Color key
-1 0 1
NTC_72h_4
NTC_72h_3
NTC_72h_2
NTC_72h_1
si-PKP2_72h_3
si-PKP2_72h_2
si-PKP2_72h_1
NS_6d_4
NS_6d_3
NS_6d_1
NS_6d_2
sh-PKP2_6d_2
sh-PKP2_6d_1
sh-PKP2_6d_3
Marker genes
G1/S phases
CCNB2
CDK1
CDKN3
DBF4
FBXO5
KIF15
MNAT1
STIL
TOP2A
S/G2 phases
BRCA2
CCNB1
CASP8AP2
EXO1
EZH2
HNRNPD
KPNA2
PRC1
RACGAP1
G2/M phases
AURKA
CCNA2
CENPF
CENPI
HMMR
KIF11
MKI67
PLK1
TPX2
XPO1
M phase
ATRX
BUB1
CDC20
CENPE
KIF20B
KIF23
KIF4A
NDC80
NEK2
NUP98
ODC1
PBK
PLK4
PTTG1
SMC4
STMN1
TACC3
TMPO
TTK
LMNB1
MAD2L1
CDC27
b
Color key
-1 0 1
Upregulated in senescent cells
CDKN1A
CDKN2A
TUSC2
ZNHIT1
ICAM1
TNFRSF1A
TNFRSF10C
BAX
ERF
SQSTM1
CREG1
SPHK1
CDK4
SLC39A3
Downregulated in senescent cells
CDKN1B
CCNE1
HMGB1
HMGB2
LMNB1
c
Unmarked NTC
Q1-UL 0%
Q1-UR 0.3%
P1 0.8%
Q1-LL 98.4%
Q1-LR 1.3%
Marked NTC
Q1-UL 0%
Q1-UR 0.2%
P1 1.1%
Q1-LL 98.1%
Q1-LR 1.7%
Marked si-PKP2
Q1-UL 0%
Q1-UR 0.3%
P1 2.1%
Q1-LL 98.4%
Q1-LR 2.9%
SSC-A
FSC-A
10
5,000,000
10,000,000
16,777,215
0
d
Unmarked Ctrl
Q1-UL 0%
Q1-UR 0.3%
P1 2,1%
Q1-LL 97.1%
Q1-LR 2.6%
Marked MCM
Q1-UL 0%
Q1-UR 0.3%
P1 3.5%
Q1-LL 95.7%
Q1-LR 4.0%
Marked Ctrl
Q1-UL 0%
Q1-UR 0.2%
P1 1,7%
Q1-LL 97.8%
Q1-LR 2.1%
Marked MCM+OE_PKP2
Q1-UL 0%
Q1-UR 0.2%
P1 1.5%
Q1-LL 98.0%
Q1-LR 1.8%
e
PKCα
NTC
si-PKP2
100 µm
PKCα+nuclei (DAPI)
PKCα
Ctrl
MCM
MCM+ OE_PKP2
PKCα+nuclei (DAPI)
f
FITC
DAPI
Overlap DAPI-FITC
Overlap FITC-DAPI

Supplementary Figure S6. Heatmaps of microarray data showing (a) cell cycle-specific and (b) senescence-associated gene expression patterns in human adipocytes with impaired PKP2 levels lasting 3 (si-PKP2) or 6 (sh-PKP2) days and respective controls. Scatter dot plots show randomly selected images and percent of senescence-associated β-galactosidase (SA-β-gal) active adipocytes under (c) siRNA-mediated PKP2 silencing, and (d) responding to MCM and complemented with a plasmid coding for PKP2 (OE_PKP2), as provided by a BD Accuri C6 flow cytometer (see also in Figure 6e). S/FSC stands for side/forward scatter measures. Below, (e) randomly selected 20x immunofluorescent images show the (f) staining of PKCα (FITC-green) in adipocytes' nuclei (DAPI-blue signal). Results assessed by means of this approach are represented as % overlap in Figure 6i, and show results assessed in one experiment (n=4 biological replicates/group).

REFERENCES

1. Ambele, M. A., Dessels, C., Durandt, C. & Pepper, M. S. Genome-wide analysis of gene expression during adipogenesis in human adipose-derived stromal cells reveals novel patterns of gene expression during adipocyte differentiation. *Stem Cell Res.* **16**, 725–734 (2016).
2. Shen, J. X. *et al.* 3D Adipose Tissue Culture Links the Organotypic Microenvironment to Improved Adipogenesis. *Adv. Sci. (Weinheim, Baden-Wurttemberg, Ger.* **8**, e2100106 (2021).
3. O'Hara, A., Lim, F. L., Mazzatti, D. J. & Trayhurn, P. Microarray analysis identifies matrix metalloproteinases (MMPs) as key genes whose expression is up-regulated in human adipocytes by macrophage-conditioned medium. *Pflugers Arch. Eur. J. Physiol.* **458**, 725–734 (2009).
4. Emont, M. P. *et al.* A single-cell atlas of human and mouse white adipose tissue. *Nature* **603**, 1103–1114 (2022).
5. Ortega, F. J. *et al.* Surgery-induced weight loss is associated with the downregulation of genes targeted by MicroRNAs in adipose tissue. *J. Clin. Endocrinol. Metab.* **100**, E1467–E1476 (2015).
6. Kerr, A. G., Andersson, D. P., Rydén, M., Arner, P. & Dahlman, I. Long-term changes in adipose tissue gene expression following bariatric surgery. *J. Intern. Med.* **288**, 219–233 (2020).
7. Larsen, T. M. *et al.* Diets with high or low protein content and glycemic index for weight-loss maintenance. *N. Engl. J. Med.* **363**, 2102–2113 (2010).
8. Vijay, J. *et al.* Single-cell analysis of human adipose tissue identifies depot and disease specific cell types. *Nat. Metab.* **2**, 97–109 (2020).
9. Norreen-Thorsen, M. *et al.* A human adipose tissue cell-type transcriptome atlas. *Cell Rep.* **40**, 111046 (2022).
