# Supplementary material for: Impaired Plakophilin-2 in obesity breaks cell cycle dynamics to breed adipocyte senescence
Source: Nat Commun. 2023 Aug 22;14:5106. doi: 10.1038/s41467-023-40596-0 (PMC10444784; doi:10.1038/s41467-023-40596-0)
Supplement: Supplementary file 3 — Reporting Summary [file 41467_2023_40596_MOESM3_ESM.pdf]

nature portfolio

Corresponding author(s): Francisco J Ortega

Last updated by author(s): Jul 12, 2023

Reporting Summary

Nature Portfolio wishes to improve the reproducibility of the work that we publish. This form provides structure for consistency and transparency in reporting. For further information on Nature Portfolio policies, see our Editorial Policies and the Editorial Policy Checklist.

Statistics

For all statistical analyses, confirm that the following items are present in the figure legend, table legend, main text, or Methods section.

n/a	Confirmed	
☐	☒	The exact sample size (*n*) for each experimental group/condition, given as a discrete number and unit of measurement
☐	☒	A statement on whether measurements were taken from distinct samples or whether the same sample was measured repeatedly
☐	☒	The statistical test(s) used AND whether they are one- or two-sided<br>*Only common tests should be described solely by name; describe more complex techniques in the Methods section.*
☐	☒	A description of all covariates tested
☐	☒	A description of any assumptions or corrections, such as tests of normality and adjustment for multiple comparisons
☐	☒	A full description of the statistical parameters including central tendency (e.g. means) or other basic estimates (e.g. regression coefficient) AND variation (e.g. standard deviation) or associated estimates of uncertainty (e.g. confidence intervals)
☐	☒	For null hypothesis testing, the test statistic (e.g. *F*, *t*, *r*) with confidence intervals, effect sizes, degrees of freedom and *P* value noted<br>*Give P values as exact values whenever suitable.*
☒	☐	For Bayesian analysis, information on the choice of priors and Markov chain Monte Carlo settings
☒	☐	For hierarchical and complex designs, identification of the appropriate level for tests and full reporting of outcomes
☐	☒	Estimates of effect sizes (e.g. Cohen's *d*, Pearson's *r*), indicating how they were calculated

Our web collection on statistics for biologists contains articles on many of the points above.

Software and code

Policy information about availability of computer code

Data collection	Samples and subsidiary data from human participants were facilitated by the IDIBGI Biobank (Biobanc IDIBGI, B.0000872), integrated in the Spanish National Biobanks Network. For human data collection, the IDIBGI Biobank uses the commercial and custom code software NorayBanks Advanced v.3.40.2205.0209 (NorayBio; Biscay, Spain).
Data analysis	For microarrays analyses, R programming (v.3.6.0) was used, together with different packages from Bioconductor (v.3.10) and the Comprehensive R Archive Network (CRAN; https://cran.r-project.org). Clariom_S_Human array CEL files were analysed for differential gene expression using Transcriptome Analysis Console (v.TAC 4.0) software (Affymetrix) to explore changes affecting transcriptome profiles between experimental groups. Heat maps representing z-scores were based on gene-counts for each sample using the package Bioinfokit (2.0.8) for Python. Three collections of gene sets were evaluated: Hallmark, C2 (containing all curated gene sets), and C5, which includes the gene ontology (GO) biological process. Pathways diagrams were performed using Enrichment Map application in Cytoscape (v.3.7.2). The lists of probes were also uploaded into the Ingenuity Pathways Analysis (IPA) v.8.7 software (Ingenuity System; Inc., http://www.ingenuity.com), and the 'Core Analysis' function included in IPA was used to interpret the results in the context of biological pathways and networks. In addition, we used Metascape (https://www.metascape.org) to create an interactome analysis to complement IPA results. For proteomics, peptide spectrum matching and label-free quantitation were performed using Spectra identification software DIA-NN for DIA data and a library-free search against the Human Uniprot.org database (20,407 Swiss-Prot entries, April 2023). Functional enrichment analysis of differentially abundant proteins was performed using MetaboAnalyst 5.0 and g:Profiler (version e109_eg56_p17_1d3191d) with g:SCS multiple testing correction method applying significance threshold of 0.05. Other statistical analyses were performed with the SPSS software v.23 (IBM, Inc.), GraphPad Prism v.5.0 (GraphPad Software Inc.), and also with R Statistical Software (http://www.r-project.org/). A flow cytometer BD Accuri™ C6 Plus Flow Cytometer (BD Biosciences) and the BD Accuri™ C6 software (v1.0.264.21) were employed to evaluate the percentage of SA-β-gal-positive cells. The Nikon's Flagship NIS-Elements Package software (version 4.2) was used for image analysis. Fluorescein 5-isothiocyanate (FITC) staining in nuclei was assessed by FIJI (https://fiji.sc/) software (v1.53t). Optical densities of the

immunoreactive bands were measured using ImageJ analysis software (v.1.53k).

For manuscripts utilizing custom algorithms or software that are central to the research but not yet described in published literature, software must be made available to editors and reviewers. We strongly encourage code deposition in a community repository (e.g. GitHub). See the Nature Portfolio guidelines for submitting code & software for further information.

Data

Policy information about availability of data

All manuscripts must include a data availability statement. This statement should provide the following information, where applicable:

- Accession codes, unique identifiers, or web links for publicly available datasets
- A description of any restrictions on data availability
- For clinical datasets or third party data, please ensure that the statement adheres to our policy

The data that support the findings of this study has been deposited in public data repositories, and/or is presented as Supplementary Information with the manuscript. The microarrays data generated have been deposited in the GEO repository under accession codes GSE182231 and GSE182229. The full list of mass spectrometry settings has been uploaded along with proteomics raw data to the ProteomeXchange Consortium (PXD042110). Additional datasets used during this research are publicly available in the GEO repository under the following accession codes: GSE53378 (PMID: 26252355), GSE199063 (PMID: 32406570), GSE95640 (PMID: 21105792), GSE77532 (PMID: 27108396), GSE186519 (PMID: 34165908), GSE14312 (PMID: 19585142), and GSE135776 (PMID: 32066997). The source data underlying Figures and Supplementary Figures and Tables contained in this article is also provided as a Source Data file, and/or uploaded to Figshare.

Research involving human participants, their data, or biological material

Policy information about studies with human participants or human data. See also policy information about sex, gender (identity/presentation), and sexual orientation and race, ethnicity and racism.

Reporting on sex and gender	For this study, we analysed abdominal biopsies of adipose tissue from two transversal/longitudinal human patient cohorts. Cohort 1 included 24 women with a body mass index up to 25 kg/m2 as controls, and 20 age-matched morbidly obese women followed for an average of ~2 years after bariatric surgery (Roux-en-Y hepaticojejunostomy), which resulted in pronounced loss of fat mass and metabolic improvement. Men were not available in this dataset. Cohort 2 consisted of 219 individuals (28% men) with a wide range of weight and glucose tolerance. Human samples were handled in accordance with the standard operating procedures and the approval of the Ethics, External Scientific and FATBANK Internal Scientific Committees. Samples and subsidiary data from human participants were facilitated by the IDIBGI Biobank, integrated in the Spanish National Biobanks Network. All subjects gave written informed consent after the purpose of the study with code number B.0000872 was explained to them. Subcutaneous adipose tissue biopsies were also obtained from patients undergoing the implantable cardioverter defibrillator procedure. We analysed fat-derived stromal cells isolated from 5 patients carrying a pathogenic mutation in PKP2, and 5 non-PKP2-mutated patients, as previously described in reference #54. The study complies with the Declaration of Helsinki and was approved by "Istituto Europeo di Oncologia e Centro Cardiologico Monzino IRCCS" Ethics Committee (CCM1072-03/07/2019).
Reporting on race, ethnicity, or other socially relevant groupings	All subjects were of Caucasian origin.
Population characteristics	Subcutaneous adipose tissue was obtained by biopsy at the mesogastric level in obese women during (49 ± 9 yrs) and ~2 years after bariatric surgery (51 ± 9 yrs), and in 24 age-matched women without obesity (45 ± 5 yrs) following other elective surgical procedures. An independent sample of ~220 subjects aged between 20 and 80 yrs (47 ± 11 yrs; 78% women), was enrolled for a comprehensive assessment of the relationship between adipose PKP2 and clinical and biochemical parameters. Subcutaneous adipose tissue biopsies were also obtained from 10 patients with ARVC (43 ± 16 yrs; 30% women) undergoing the implantable cardioverter defibrillator procedure.
Recruitment	Our cohorts were recruited at the Department of Diabetes, Endocrinology and Nutrition and the Department of Surgery of the Hospital "Dr Josep Trueta" of Girona (Girona, Spain). All subjects provided written informed consent before entering the study, were of Caucasian origin, and reported that their body weight had been stable for at least three months before entering the study. No systemic disease other than type 2 diabetes and/or obesity was reported. All participants were free of any infections in the previous month before entering the study. Liver and thyroid dysfunction were specifically excluded by biochemical work-up. Other exclusion criteria included: 1) clinically significant hepatic, neurological, or other major systemic disease, including malignancy, 2) history of drug or alcohol abuse, or serum transaminase activity more than twice the upper limit of normal, 3) elevated serum creatinine concentration, 4) acute major cardiovascular event in the previous 6 months, 5) acute illnesses and current evidence of high grade chronic inflammatory or infective diseases, and 6) mental illness rendering the subjects unable to understand the nature, scope, and possible consequences of this study. Adipose tissue samples from men and women without severe obesity were collected during elective surgical procedures (e.g., cholecystectomy and surgery of abdominal hernia). Biopsies of adipose tissue were obtained from morbid obese participants during bariatric surgery (Roux-en-Y hepaticojejunostomy). Abdominal subcutaneous and visceral (from greater omentum) adipose tissue was collected at the beginning of surgical interventions. Subcutaneous samples of fat from morbid obese patients upon ~2 years of surgery-induced weight loss were acquired by fine-needle aspiration in the morning after an overnight fast. Subcutaneous adipose tissue biopsies were also obtained from patients undergoing the implantable cardioverter defibrillator procedure in 5 patients carrying a pathogenic mutation in PKP2, and 5 non-PKP2-mutated patients, as previously described in reference #54. The analysis complies with the Declaration of Helsinki and was approved by "Istituto Europeo di Oncologia e Centro Cardiologico Monzino IRCCS" Ethics Committee (CCM1072-03/07/2019). No self-selection bias is considered, yet, because our observations in bulk adipose tissue and adipose-derived cell samples were not population-based, but rather patient-centered, we cannot exclude that the relationship with obesity and other clinical characteristics relies on the characteristics of our volunteers. Nevertheless, experiments in vitro, together with the systematic scrutiny of multiple human datasets and observations made in obese subjects following weight loss, support to a large extent the connexions postulated in our article.

Ethics oversight

Samples and data from patients included in this study were provided by the FATBANK platform, promoted by the CIBEROBN and coordinated by the IDIBGI Biobank (Biobanc IDIBGI, B.0000872), integrated in the Spanish National Biobanks Network. All samples were processed following standard operating procedures with the appropriate approval of the Ethics, External Scientific and FATBANK Internal Scientific Committees. Subcutaneous adipose tissue biopsies were obtained from patients undergoing the implantable cardioverter defibrillator procedure. This study complies with the Declaration of Helsinki and was approved by "Istituto Europeo di Oncologia e Centro Cardiologico Monzino IRCCS" Ethics Committee (CCM1072-03/07/2019). A material transfer agreement (MTA) document was filled and signed by legal representatives of the organizations involved into the transfer of this material, and the "Recipient" (IDIBGI) agreed to use it for research purposes only, without major modifications nor commercial applications.

Note that full information on the approval of the study protocol must also be provided in the manuscript.

Field-specific reporting

Please select the one below that is the best fit for your research. If you are not sure, read the appropriate sections before making your selection.

☒ Life sciences ☐ Behavioural & social sciences ☐ Ecological, evolutionary & environmental sciences

For a reference copy of the document with all sections, see nature.com/documents/nr-reporting-summary-flat.pdf

Life sciences study design

All studies must disclose on these points even when the disclosure is negative.

Sample size

The availability of samples and patient characteristics determined the total sample size.

Data exclusions

No data were excluded from the analyses.

Replication

We confirm for all experiments that all attempts at replication were successful. The reproducibility of our experimental findings in differentiating human and mouse adipocytes, and lines of evidence taken from other studies led us to confirm dynamic adaptations of PKP2 during adipogenesis. To validate longitudinal changes affecting adipose PKP2 gene expression in human cohorts, we checked results from three independent next-generation sequencing and microarray analyses, as depicted in the manuscript. Several vials of commercially available cryopreserved preadipocytes from subcutaneous and omental adipose tissues were purchased, plated in culture, stimulated with adipogenic conditions, and treated as explained in the manuscript. At least 4 biological replicates (i.e., wells of rounded fully-differentiated preadipocytes in confluence with large lipid droplets apparent in the cytoplasm) were included for all experiments in vitro. To validate our transcriptional data, we ruled out multiple assays together with tests for cell cycle dynamics and cellular senescence. Our comprehensive pipeline in different cultures of primary human adipocytes allowed us to sequentially filter and validate our preselected biomarkers throughout a set of experimental approaches. Finally, we cultured and studied mesenchymal stromal cells obtained from Subcutaneous adipose tissue biopsies obtained in patients undergoing the implantable cardioverter defibrillator procedure. Digested biopic samples and isolated stromal cells were studied as a dry pellet, and seed in culture and differentiated (or not) into adipocytes. Four biological replicates were studied for each cell type and subject.

Randomization

Patient grouping was strictly based on clinical characteristics. Mice grouping was based on their genetic condition (PKP2+/- or wild-type). During in vitro experiments, cell culture plates were distributed as to guaranteed that treated (e.g. MCM) and/or modified (PKP2-knocked down adipocytes) cells and their respective controls were comparable in each experimental setting/method (e.g., IF, gene expression, western blot, etc.), and that in the same plates cohabited cells treated/modified and controls.

Blinding

All human samples were coded and analyses were performed once all data had been collected. During data collection, investigators were blinded to group allocation, as all samples are encoded. During data analysis, once results were located in the general database, blinding was not possible. As subjects grouping was strictly based on clinical characteristics (i.e., paired samples pre-post bariatric surgery, obesity (as defined by a BMI≥30 kg/m2), and/or impaired glucose tolerance), we argue that blinding was not relevant to this study.

Reporting for specific materials, systems and methods

We require information from authors about some types of materials, experimental systems and methods used in many studies. Here, indicate whether each material, system or method listed is relevant to your study. If you are not sure if a list item applies to your research, read the appropriate section before selecting a response.

Materials & experimental systems

n/a	Involved in the study	
☐	☒	Antibodies
☐	☒	Eukaryotic cell lines
☒	☐	Palaeontology and archaeology
☐	☒	Animals and other organisms
☒	☐	Clinical data
☒	☐	Dual use research of concern
☒	☐	Plants

Methods

n/a	Involved in the study	
☒	☐	ChIP-seq
☐	☒	Flow cytometry
☒	☐	MRI-based neuroimaging

Antibodies

Antibodies used	For IF: Primary mouse monoclonal antibody against PKP2 (sc-393711, clone C1, Santa Cruz Biotechnology, Inc.), rabbit monoclonal anti-Cyclin D1 (#55506, clone E3P5S, Cell Signalling), and rabbit polyclonal anti-PKCα (#2056). Alexa Fluor 546™ Goat anti-rabbit (#A-11081, Invitrogen), Alexa Fluor™ 594 secondary antibody (ab150116, Abcam), and goat anti-Rabbit IgG (H+L) Highly Cross-Adsorbed Secondary Antibody Alexa Fluor™ 488 (#A-11034, Invitrogen) as secondary antibodies.<br><br>For WB: Mouse monoclonal anti-PKP2 (sc-393711, clone C1), hFAB™ Rhodamine Anti-Tubulin Primary Antibody (#12004166) from Bio-Rad, mouse monoclonal anti-β-actin (sc-47778, clone C4), and rabbit polyclonal anti-FAS (sc-20140) from Santa Cruz Biotechnology, Inc., anti-PAPPA (AF2487, R&D Systems), and anti-phospho-p38 MAPK (Thr180/Tyr182) (#9215, clone 3D7), anti-p38 MAPK (#9212), anti-phospho-Akt (Ser473) (#9271), anti-Akt (#9272) and anti-Cyclin D1 (#2978, clone 92G2C8) from Cell Signalling. Following incubation with the primary antibody, the membranes were washed and incubated with the appropriate IgG-HRP-conjugated secondary antibody (i.e., polyclonal rabbit anti-mouse (#P0260) from Dako, anti-rabbit IgG HRP-linked antibody (#7074) provided by Cell Signalling, or goat IgG HRP-conjugated antibody (#HAF017) from R&D Systems).
Validation	Anti-PKP2 (#sc-393711, clone C1): fluorescent western blot analysis of PKP2 expression in HepG2 whole cell lysate, western blot analysis in NIH/3T3, U-87 MG whole cell lysates, HeLa nuclear extract and in non-transfected (sc-117752), and human PKP2 transfected (sc-372976 293T) whole cell lysates and HeLa nuclear extract. anti-CyclinD1 (#55506, clone E3P5S): immunofluorescent analysis of SH-SY5Y cells and 293T cells. anti-β-actin (#sc-47778, clone C4): western blot analysis in Jurkat, HeLa and A-431 whole cell lysates. anti-PKCα (#2056): western blot analysis of extracts of HeLa, COS, C6 and NIH/3T3 cells and analysis of extracts of Baculovirus expressed PKC isoforms demonstrating the isoform-specificity of PKCα Antibody. anti-tubulin (#12004166): not specified. anti-FAS (#sc-20140): western blot analysis in HeLa, 3T3-L1, Hep G2, A549 and PC-3 whole cell lysates and mouse liver tissue extract. anti-PAPPA (#AF2487): western blot of human pregnant sera lysates. anti-phospho-p38 MAPK (Thr180/Tyr182) (#9215, clone 3D7): western blot analysis of extracts from NIH/3T3 cells treated with PDGF and UV, extracts from Jurkat, C6, NIH/3T3 and COS cells. anti-p38 MAPK (#9212): western blot analysis of extracts from HeLa cells, transfected with 100 nM SignalSilence® Control siRNA (Unconjugated) #6568 (-), SignalSilence® p38 MAPK siRNA I #6564 (+) or SignalSilence® p38 MAPK siRNA II (+), and western blot analysis of extracts from C6 cells, untreated or anisomycin-treated, and NIH/3T3 cells, untreated or UV-treated. anti-phospho-Akt (Ser473) (#9271): Western blot analysis of extracts from NIH/3T3 cells, untreated or treated with PDGF, wortmannin, LY294002, rapamycin or PD98059 and western blot analysis of immunoprecipitated Akt from 293 cells transiently transfected with HA-tagged Akt (WT), HA-tagged K179A mutant Akt and HA-tagged K179A/S473A mutant Akt. anti-Akt (#9272): western blot analysis of extracts from CHO cells, transfected with non-targeted (-) or SignalSilence® Akt siRNA I (+) siRNA, western blot analysis of extracts from HeLa cells, transfected with 100 nM SignalSilence® Control siRNA (Fluorescein Conjugate) #6201 (-) or SignalSilence® Akt siRNA I (+) and western blot analysis of extracts from NIH/3T3 cells, untreated or PDGF-treated (50 ng/ml). anti-CyclinD1 (#2978, clone 92G2C8): western blot analysis of extracts from MCF7, L929 and C6 cells.

Eukaryotic cell lines

Policy information about cell lines and Sex and Gender in Research

Cell line source(s)	Commercially available (Zen-Bio, Inc.) cryopreserved preadipocytes were obtained from subcutaneous and omental adipose tissues (SP-F-2 and OM-F-2, respectively) of a woman of 34 yrs and a body mass index (BMI) of 26.3 kg/m2. Subcutaneous adipocyte progenitor cells from lean (BMI<25 kg/m2) and obese (BMI>30 kg/m2) normoglycemic women (SP-F-1 and SP-F-3, respectively) of approximately same age (35-40 yrs) were also plated in culture and stimulated with adipogenic conditions. Several other vials SP-F-2 were purchased, cultured, differentiated and treated as explained in the manuscript. The human monocyte cell line THP-1 (ATCC, TIB-202) was used to obtain cell culture medias enriched in macrophage-derived pro-inflammatory cytokines. For cross-species validation purposes, the embryonic fibroblast mouse 3T3-L1 cell line (ATCC, CL-173) was cultured, differentiated and treated as explained in the manuscript.
Authentication	The THP-1 and 3T3-L1 cell lines used during this research were not authenticated in our lab. Yet, it should be noted that expression of cell-specific human/mouse gene expression biomarkers was assessed as per routine during our experiments.
Mycoplasma contamination	Tests of mycoplasma are systematically made in our cell lines by means of PCR amplification with Metabion International AG paired forward/reverse primers (F: 3'-GGCGAATGGGTGAGTAACACG-5'; R: 3'-CGGATAACGCTTGCGACTATG-5'), followed by agarose gel electrophoresis of the DNA amplification product. The lack of band (~580 bp fragment) in the corresponding agarose gel lane is acknowledged as mycoplasma-free samples. Positive and negative controls are included in each agarose gel. Purchased primary human preadipocytes "(...) are tested for viability, correct vial counts, sterility; [and are] negative for mycoplasma, HIV-1, HIV-2, hepatitis B, hepatitis C, CD31 (cell surface marker for endothelial cells)", as stated by the manufacturer (Zen-Bio, Inc.).
Commonly misidentified lines<br>(See ICLAC register)	No misidentified cell lines were used during this research.

Animals and other research organisms

Policy information about studies involving animals; ARRIVE guidelines recommended for reporting animal research, and Sex and Gender in Research

Laboratory animals	C57Bl/6 Pkp2 heterozygous knock-out mice (Pkp2+/−) were produced by Prof. Walter Birchmeier's lab, as described in Grossmann et al. (PMID: 15479741). Mice were housed in a room with controlled temperature (~20°C) with 50% humidity and a 12:12 light/dark cycle (6 a.m. – 6 p.m.). Animals were sacrificed by carbon dioxide inhalation, the external surface were sterilized with 70% ethanol and subcutaneous fat samples were collected.

Wild animals	The study did not involve wild animals.
Reporting on sex	Current findings do not apply to only one sex. Information regarding sex and age in laboratory animals used in this research was collected and is provided in Table S6.
Field-collected samples	The study did not involve samples collected from the field.
Ethics oversight	Experiments were authorized by the Italian Ministry of Health, protocol no. 249/2020-PR.

Note that full information on the approval of the study protocol must also be provided in the manuscript.

Flow Cytometry

Plots

Confirm that:

- [x] The axis labels state the marker and fluorochrome used (e.g. CD4-FITC).
- [x] The axis scales are clearly visible. Include numbers along axes only for bottom left plot of group (a 'group' is an analysis of identical markers).
- [x] All plots are contour plots with outliers or pseudocolor plots.
- [x] A numerical value for number of cells or percentage (with statistics) is provided.

Methodology

Sample preparation	Differentiated human adipocytes were incubated with Bafilomycin A1 (#54645) at 100nM for 1h at 37ºC. Then, SA-β-Gal Fluorescent Substrate (#38154) was added, mixed gently, and incubated at 37ºC for 3h. Cells were washed 3 times with 1x PBS before being trypsinized and inactivated with PBS containing 2% FBS. A flow cytometer BD Accuri™ C6 Plus Flow Cytometer (BD Biosciences) was employed to evaluate the percentage of SA-β-gal -positive cells.
Instrument	BD Accuri™ C6 Plus Flow Cytometer (BD Biosciences).
Software	BD Accuri™ C6 software (v1.0.264.21).
Cell population abundance	SA-β-gal activity was quantified by means of flow cytometry and The Senescence B-Galactosidase Activity Assay Kit (#35302, CST). Briefly, confluent cells were incubated with Bafilomycin A1 (#54645) at 100nM for 1h at 37ºC. Then, SA-β-Gal Fluorescent Substrate (#38154) was added, mixed gently, and incubated at 37ºC for 3h. Cells were washed 3 times with 1x PBS before being trypsinized and inactivated with PBS containing 2% FBS. Then, a flow cytometer BD Accuri™ C6 Plus Flow Cytometer (BD Biosciences) was employed to evaluate the percentage of SA-β-gal-positive cells. A count of 100,000 events per sample/well was assessed by means of the cytometer. After sorting, we evaluated the mature adipocyte cell population, differentiated from immature small adipocytes and cell debris based on cell size and complexity, so this population appeared shifted to the right-upper part of the SSC-A/FSC-A plot. The fully-differentiated lipid-containing mature adipocyte population assessed represented between 0.8-4% of the total sample population.
Gating strategy	The mature adipocyte relevant population was differentiated from immature small adipocytes and cell debris by size (forward) and complexity (side scattering). A negative control without SA-β-gal staining was used during our gating strategy to set up the population of interest and to subtract any unspecific signal. Then, the percentage of SA-β-gal positive mature adipocytes was assessed for each condition, and changes were compared between treated cells and respective controls. The gating strategy is exemplified in Supplementary Figures S6c and S6d, where the selected adipocyte population is shown for all conditions.

- [x] Tick this box to confirm that a figure exemplifying the gating strategy is provided in the Supplementary Information.
